# Supplementary material for: Prognostic Significance of PD-L1 Expression on Circulating Myeloid-Derived Suppressor Cells in NSCLC Patients Treated with Anti-PD-1/PD-L1 Checkpoint Inhibitors
Source: Int J Mol Sci. 2024 Nov 15;25(22):12269. doi: 10.3390/ijms252212269 (PMC11594642; doi:10.3390/ijms252212269)
Supplement: Supplementary file 1 [file ijms-25-12269-s001.zip › ijms-3280098-supplementary.pdf]

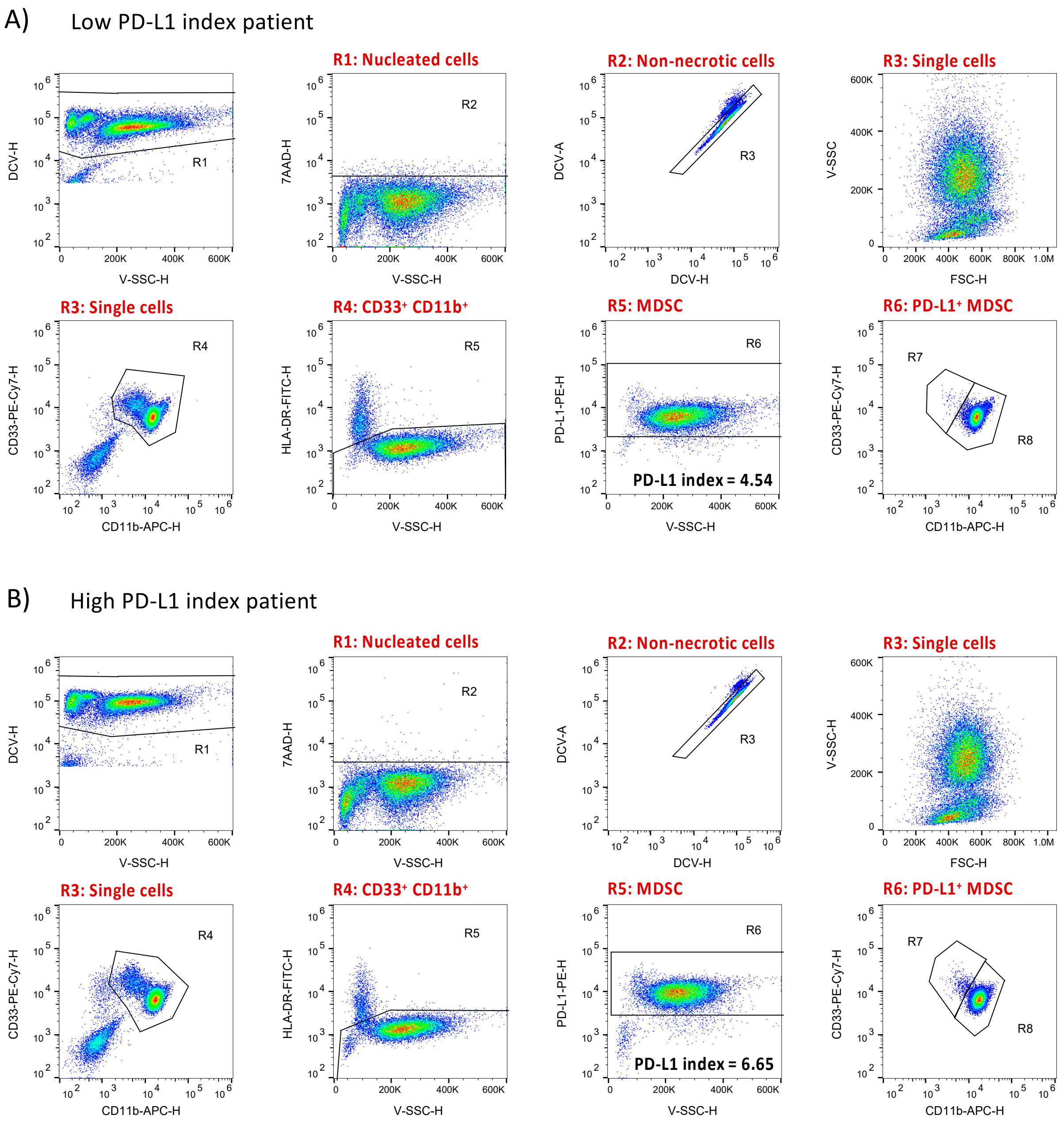
A) Low PD-L1 index patient
R1: Nucleated cells
R2: Non-necrotic cells
R3: Single cells
R3: Single cells
R4: CD33⁺ CD11b⁺
R5: MDSC
R6: PD-L1⁺ MDSC
PD-L1 index = 4.54
B) High PD-L1 index patient
R1: Nucleated cells
R2: Non-necrotic cells
R3: Single cells
R3: Single cells
R4: CD33⁺ CD11b⁺
R5: MDSC
R6: PD-L1⁺ MDSC
PD-L1 index = 6.65
DCV-H
V-SSC-H
7AAD-H
DCV-A
V-SSC
V-SSC-H
FSC-H
CD33-PE-Cy7-H
CD11b-APC-H
HLA-DR-FITC-H
PD-L1-PE-H
R1
R2
R3
R4
R5
R6
R7
R8

C)

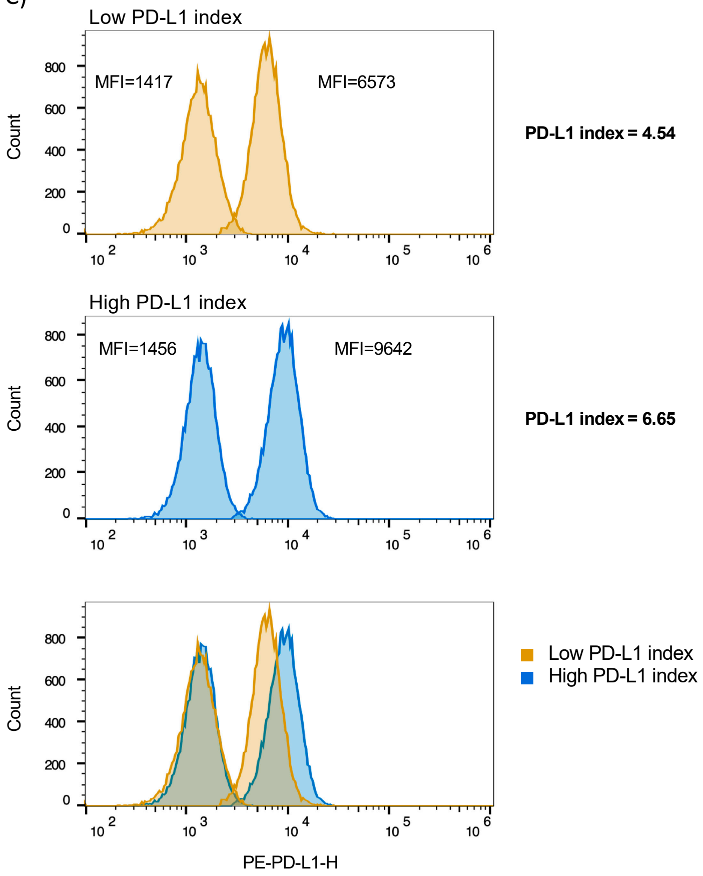

Low PD-L1 index
MFI=1417
MFI=6573
PD-L1 index = 4.54
High PD-L1 index
MFI=1456
MFI=9642
PD-L1 index = 6.65
Low PD-L1 index
High PD-L1 index
Count
PE-PD-L1-H

Figure S1. Comparison of two patients with different PD-L1 index. Representative analysis of a low PD-L1 index patient with a value of 4.54 (A) and a high PD-L1 index patient with a value of 6.65 (B). The mean fluorescence intensity (MFI) and standard deviation are used to calculate the PD-L1 index, where patients with a high PD-L1 index show a higher reactivity against the PE-PD-L1 antibody.

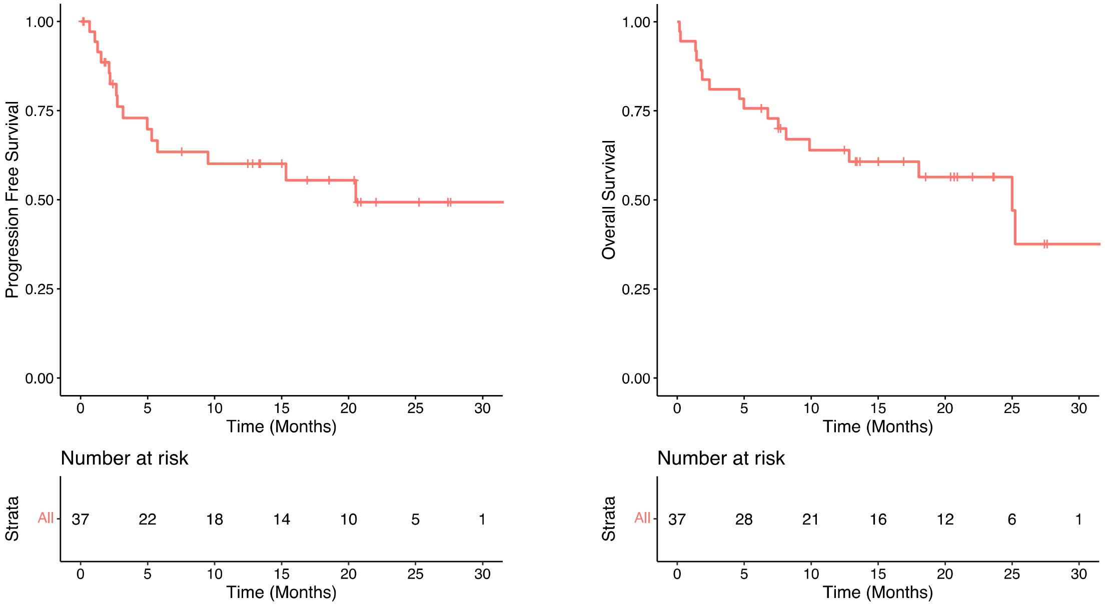

Figure S2. Kaplan-Meier plot of progression-free survival and overall survival from stage III-IV NSCLC patients.

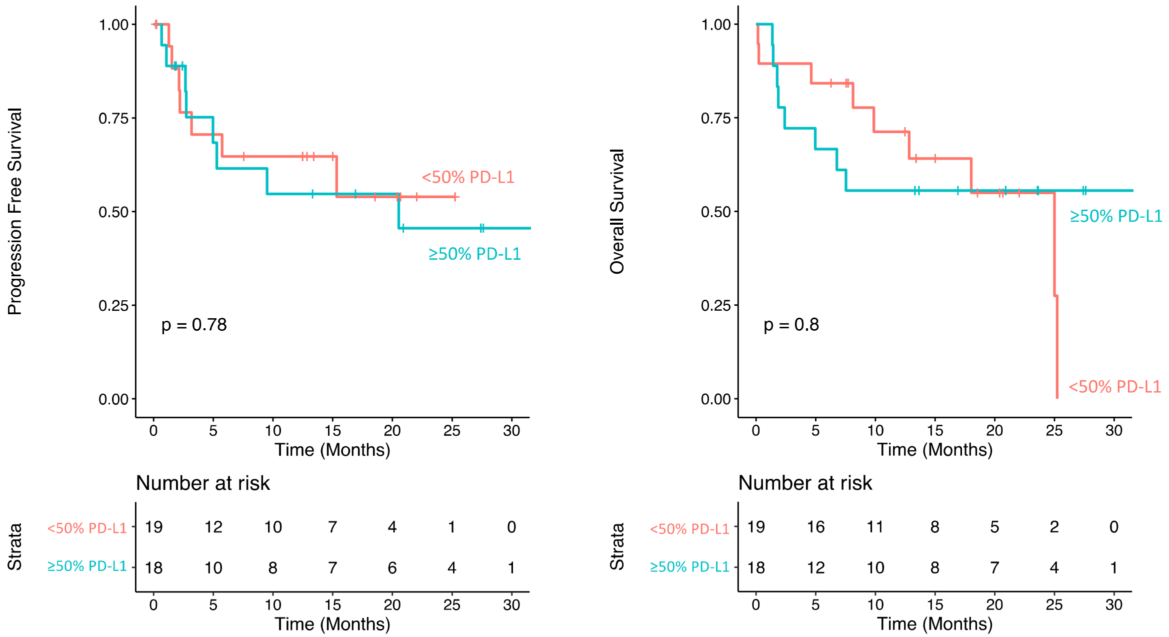

Figure S3. PD-L1 expression of tumor tissue, assessed by immunohistochemistry, did not predict PFS nor OS at baseline.

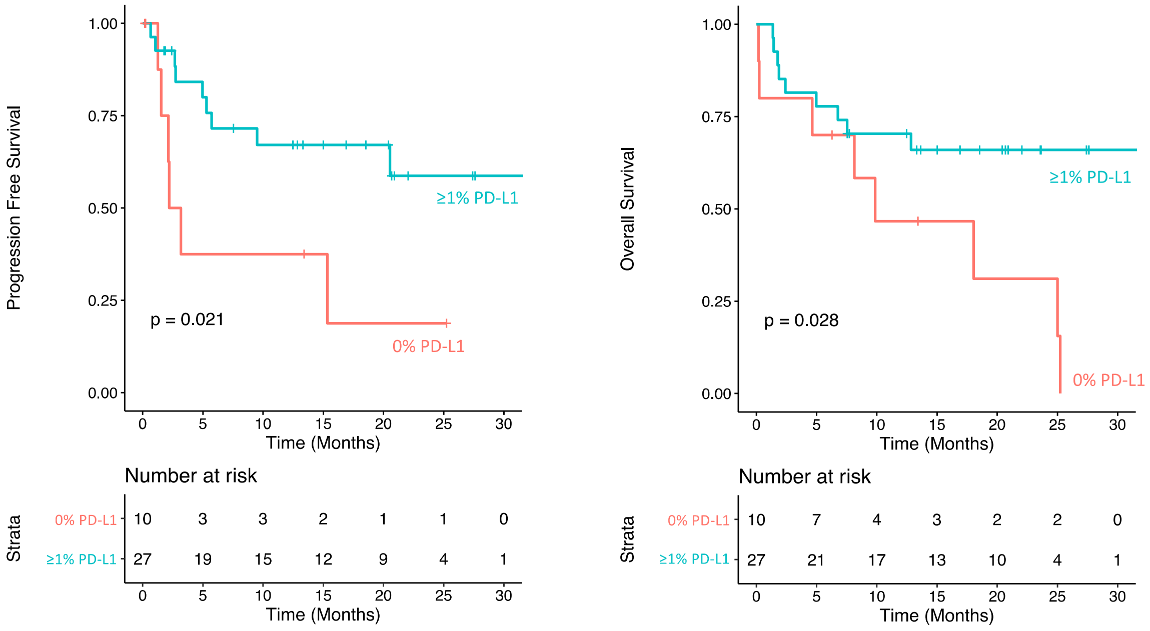

Figure S4. PD-L1 expression of tumor tissue stratified by expression and no expression predicted PFS and OS at baseline.

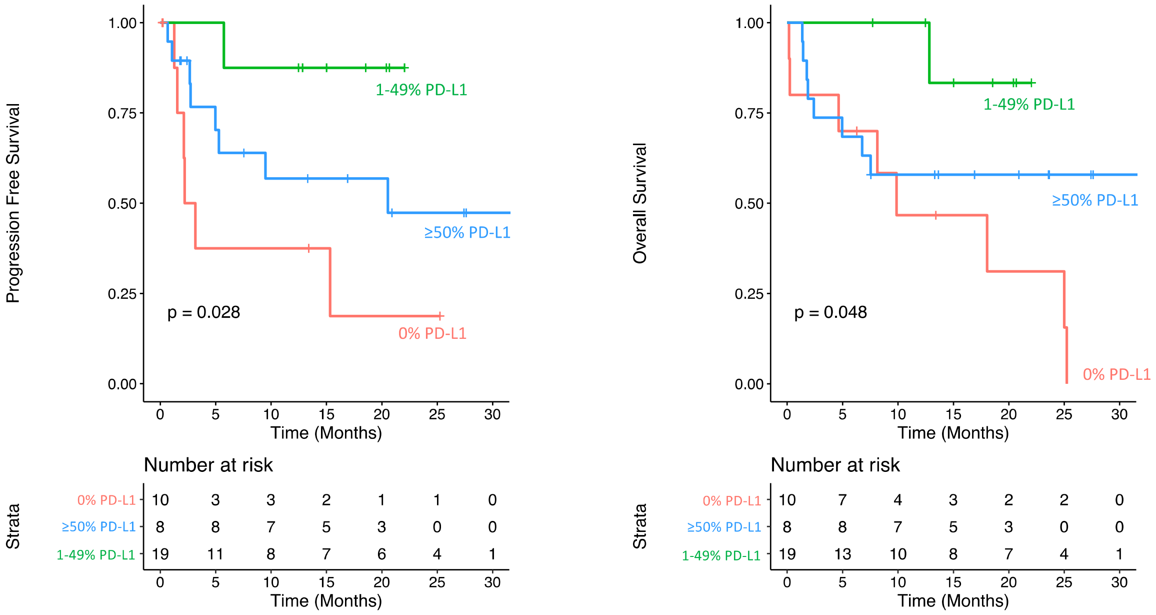

Figure S5. PD-L1 expression of tumor tissue stratified in three levels of expression.

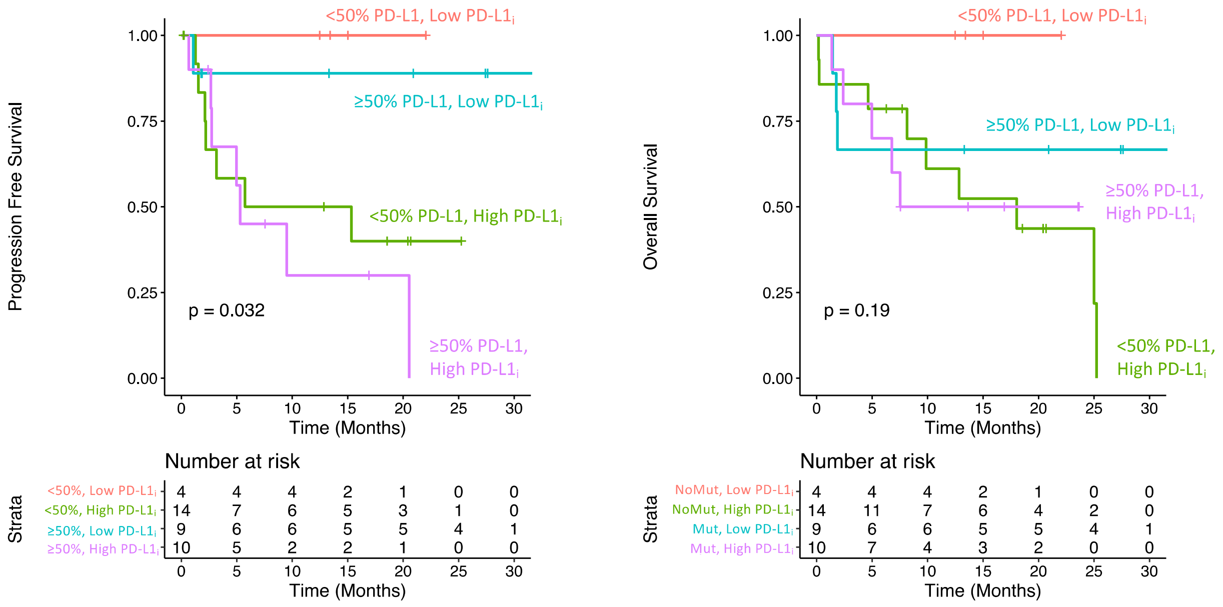

Figure S6. The combination of tumor PD-L1 expression and PD-L1 index did not improve the PFS nor OS prediction at baseline.

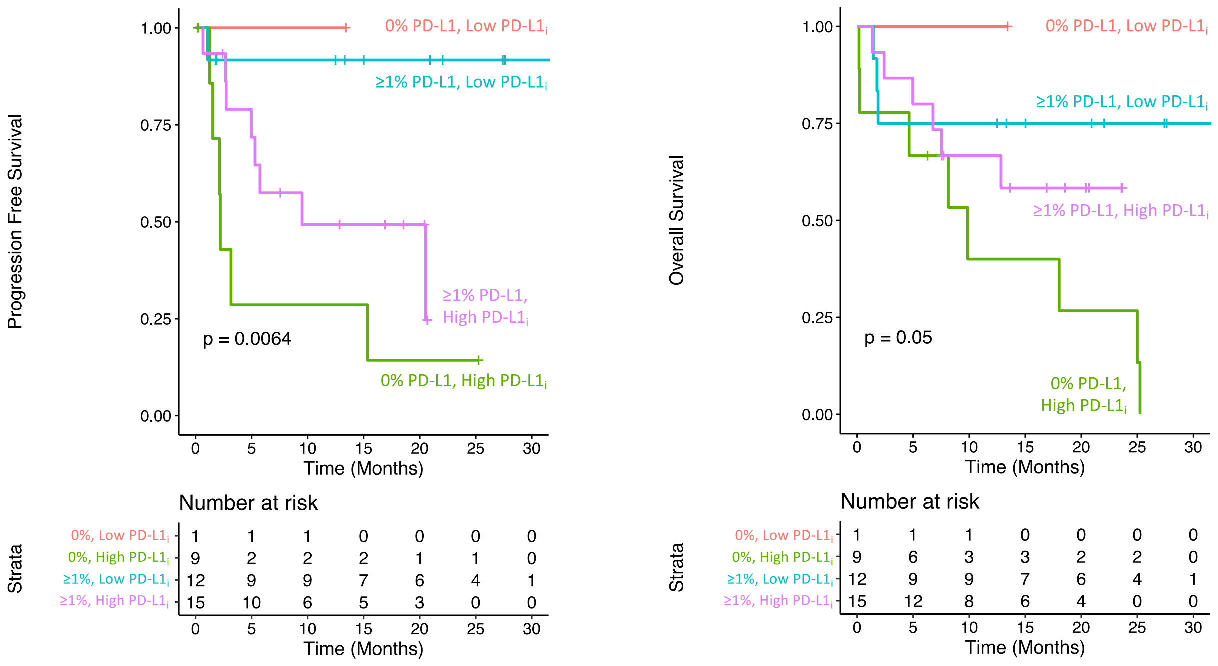

Figure S7. The combination of tumor PD-L1 expression and PD-L1 index did not improve the PFS nor OS prediction at baseline.

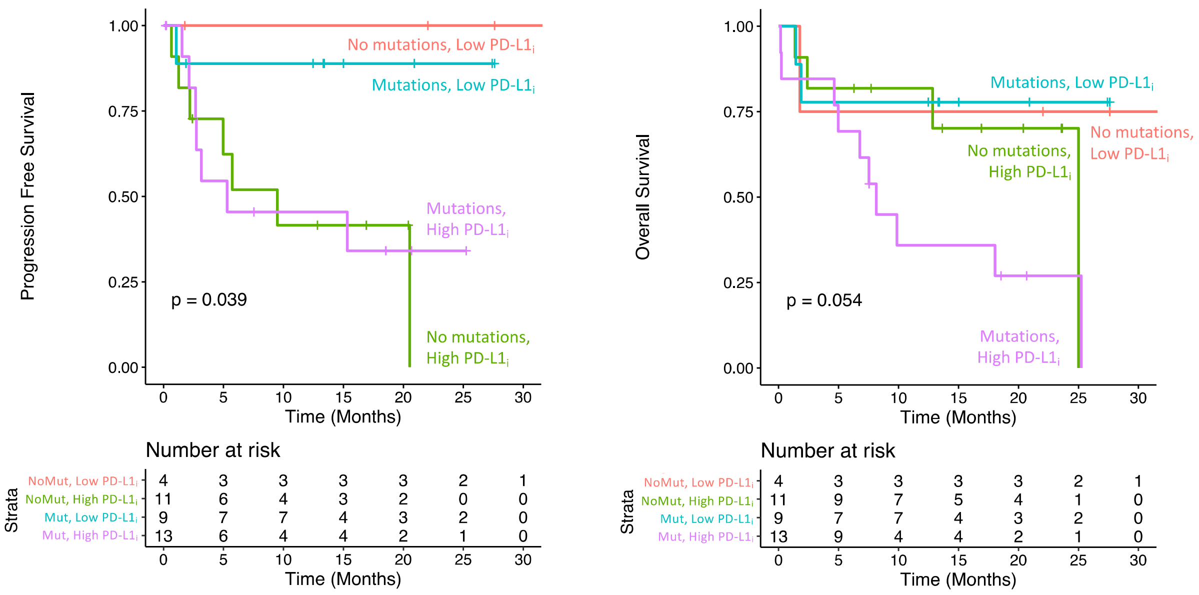

Figure S8. The presence of driver mutations combined with PD-L1 index did not improve the PFS nor OS prediction at baseline.

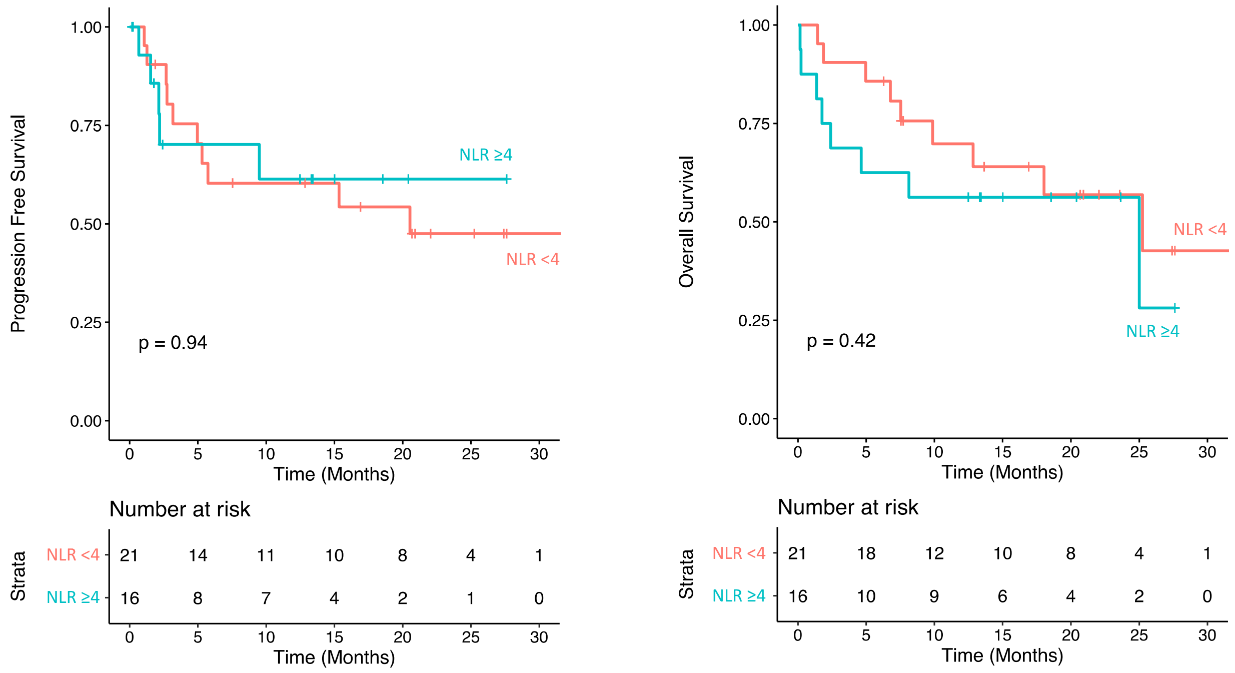

Figure S9. Neutrophil-to-lymphocyte ratio (NLR) did not predict PFS nor OS at baseline.

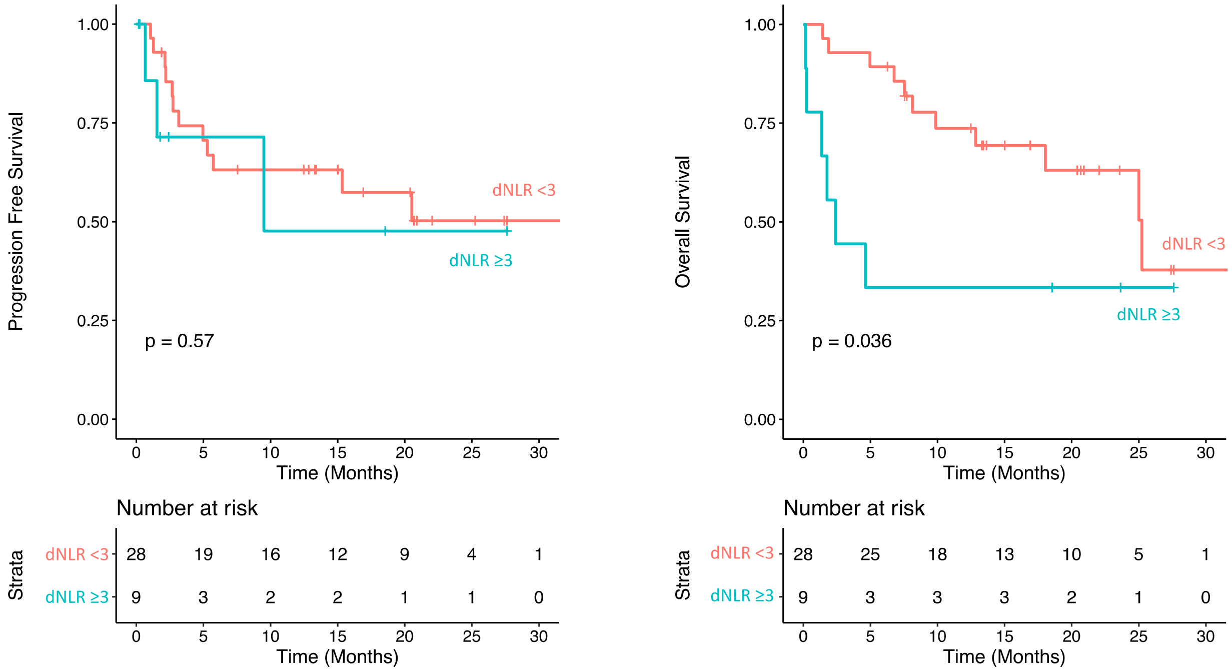

Figure S10. Derived neutrophil-to-lymphocyte ratio (dNLR) did not predict PFS nor OS at baseline.

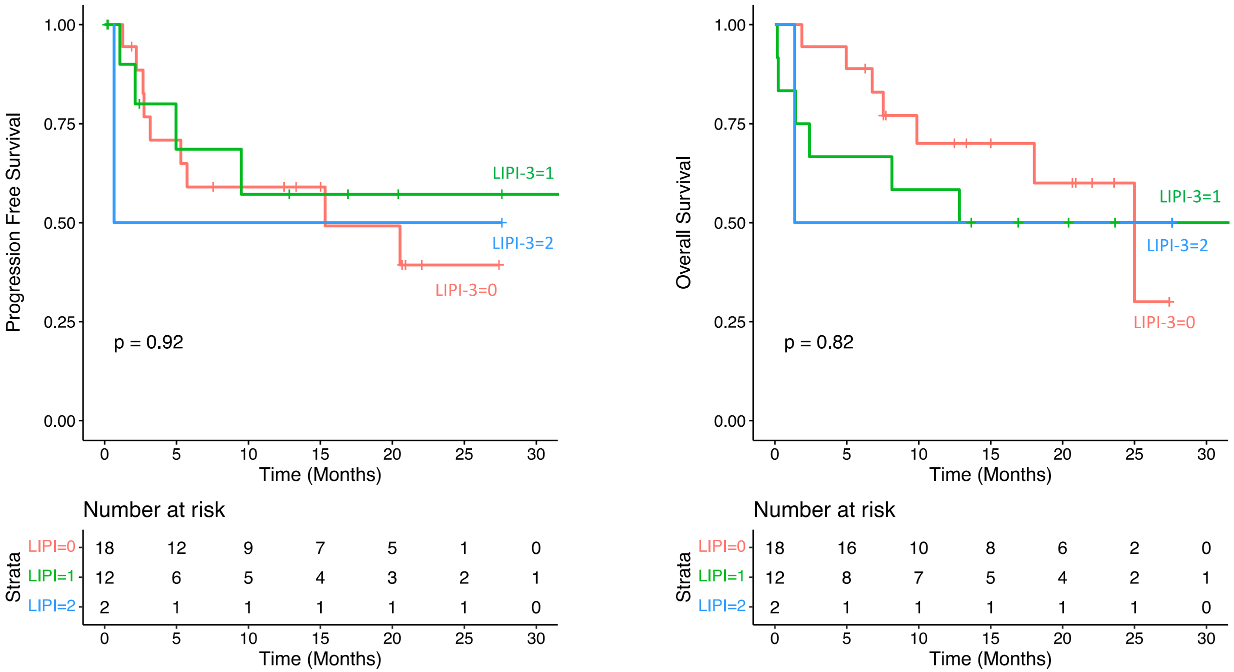

Figure S11. Lung immune prognostic index (LIPI) did not predict PFS nor OS at baseline.

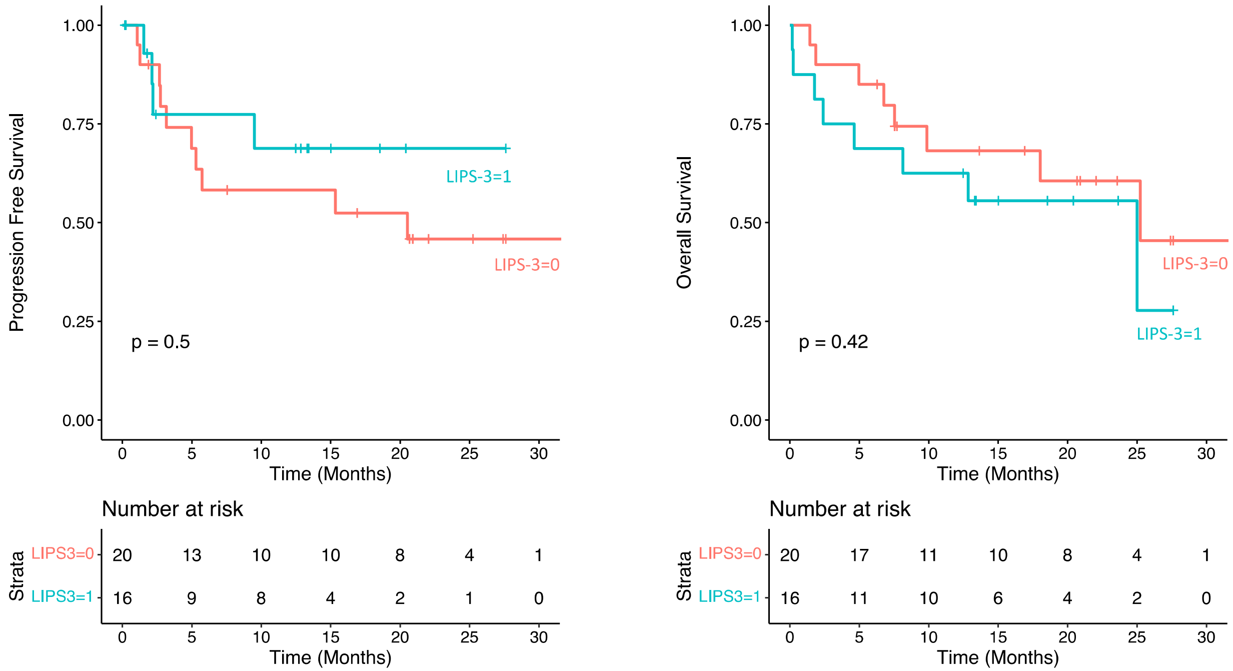

Figure S12. Lung immuno-oncology prognostic score-3 (LIPS-3) did not predict PFS nor OS at baseline.

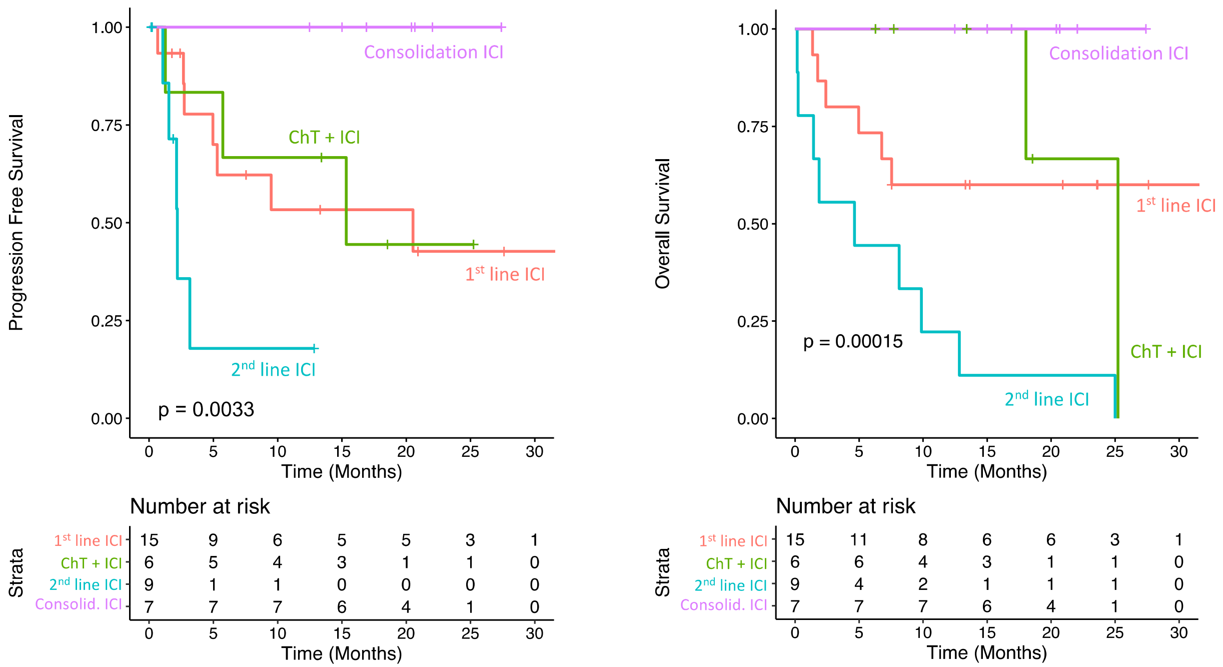

Figure S13. Treatment-strategy influence on PFS and OS. In first-line ICI and first-line chemotherapy plus ICI (ChT + ICI), pembrolizumab is administered. In second-line ICI the therapy indication is pembrolizumab, nivolumab and atezolizumab, and in consolidation ICI after chemoradiation, durvalumab is administered.

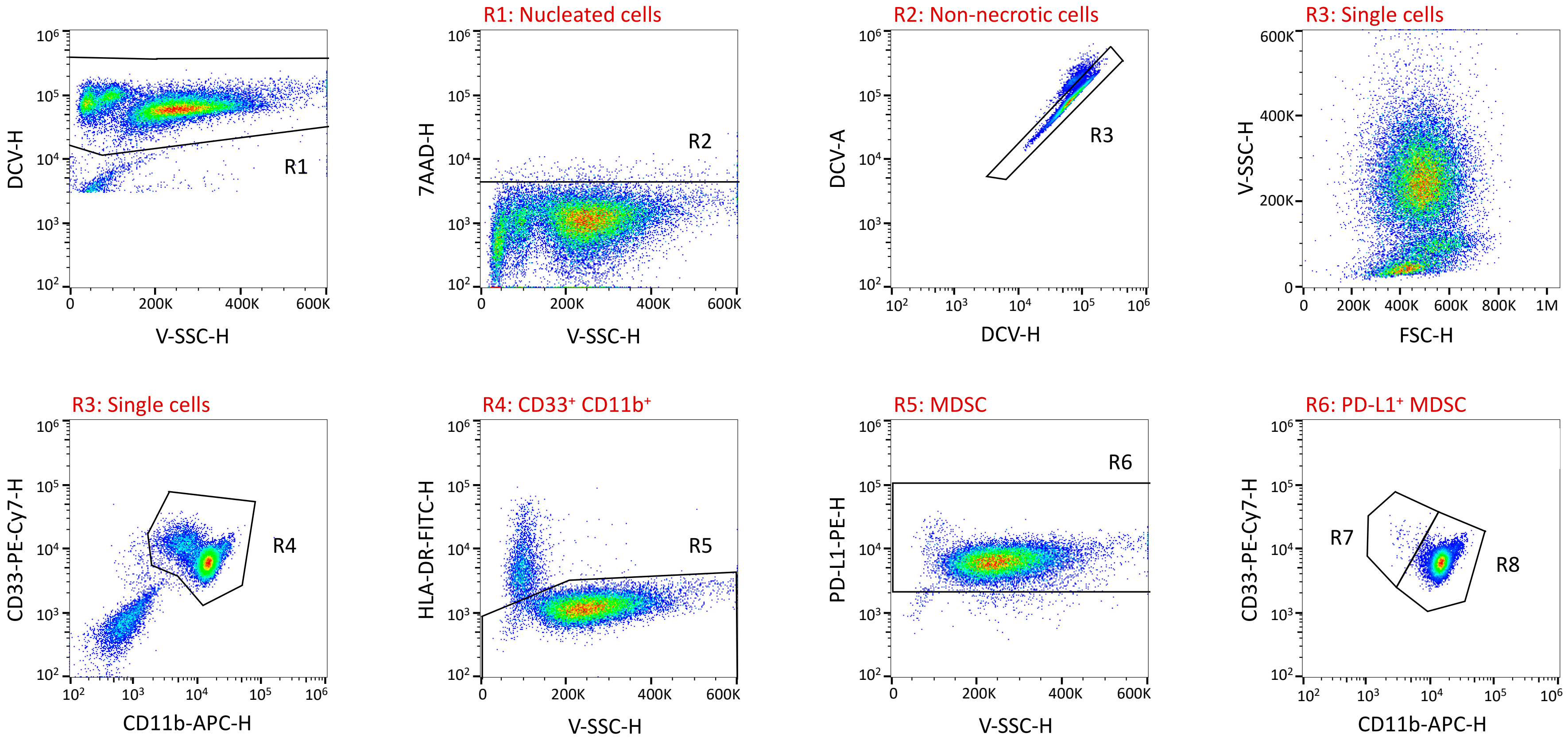

Figure S14. Flow cytometry panel and gating strategy for identification and classification of PD-L1⁺ MDSCs. Representative analysis of a peripheral blood sample from an NSCLC patient. Nucleated cells are selected based on DCV⁺ events (R1) and both necrotic cells (R2) and doublets (R3) are excluded from the analysis. CD33⁺CD11b⁺HLA-DR^-/lo events are then selected as MDSCs (R4-5). PMA-stimulated cells are highly reactive to the anti-PD-L1 monoclonal antibody and are selected as PD-L1⁺ MDSCs (R6). MDSCs can be further classified into monocytic (R7) and polymorphonuclear MDSCs (R8).

Table S1. Univariate PFS analysis

Variables			Stratified Kaplan-Meier model				Cox regression			
	Stratum value	N	PD events (%)	Censored (%)	Median (95% CI)	Log-rank (p-value)	N	Contrast	Pr > ChiSq	Hazard Ratio (95% CI)
**PD-L1 index groups**	High PD-L1 index	24	14 (58.33%)	10 (41.67%)	5.73 (2.67,20.53)	0.0042	37	PD-L1 index: High vs Low PD-L1 index	**0.0212**	11.01 (1.43,84.67)
	Low PD-L1 index	13	1 (7.69%)	12 (92.31%)	NR (NR,NR)					
**Sex**	Female	10	1 (10.00%)	9 (90.00%)	NR (20.53,NR)	0.0025	37	Sex: Male vs Female	**0.0143**	13.44 (1.68,107.53)
	Male	27	14 (51.85%)	13 (48.15%)	5.73 (2.67,NR)					
**Age**	≤68 years	19	8 (42.11%)	11 (57.89%)	20.53 (3.17,NR)	0.9048	37	Age: >68 vs ≤68 years	0.9046	1.06 (0.38,2.95)
	>68 years	18	7 (38.89%)	11 (61.11%)	15.33 (2.73,NR)					
**Smoker**	No	2	1 (50.00%)	1 (50.00%)	NR (3.17,NR)	0.9975	37	Smoker: Yes vs No	0.9975	1.00 (0.13,7.62)
	Yes	35	14 (40.00%)	21 (60.00%)	20.53 (5.30,NR)					
**Former smokers**	No	13	6 (46.15%)	7 (53.85%)	20.53 (3.17,NR)	0.9142	37	Former smokers: Yes vs No	0.9137	0.94 (0.34,2.66)
	Yes	24	9 (37.50%)	15 (62.50%)	NR (2.73,NR)					
**Current smokers**	No	26	10 (38.46%)	16 (61.54%)	NR (2.73,NR)	0.9119	37	Current smokers: Yes vs No	0.9119	1.06 (0.36,3.11)
	Yes	11	5 (45.45%)	6 (54.55%)	20.53 (1.27,NR)					
**PS (ECOG)**	0	11	4 (36.36%)	7 (63.64%)	NR (3.17,NR)	0.4236	37	Performance status (ECOG): 1,2 vs 0	0.4279	1.59 (0.50,5.01)
	1,2	26	11 (42.31%)	15 (57.69%)	15.33 (2.73,NR)					
	IV	25	13 (52.00%)	12 (48.00%)	15.33 (2.73,NR)					
**Cancer staging**	III	12	2 (16.67%)	10 (83.33%)	NR (9.50,NR)	0.0558	37	Cancer staging: IV vs III	0.0757	3.87 (0.87,17.21)
	IV	25	13 (52.00%)	12 (48.00%)	15.33 (2.73,NR)					
**Histology**	Adenocarcinoma	24	10 (41.67%)	14 (58.33%)	NR (5.30,NR)	0.4614	37	Histology: Adenocarcinoma vs Squamous cell carcinoma	0.4641	0.67 (0.23,1.97)
	Squamous cell carcinoma	13	5 (38.46%)	8 (61.54%)	NR (1.53,NR)					
**Number of metastases**	1 metastasis	17	9 (52.94%)	8 (47.06%)	15.33 (2.73,NR)	0.6687	25	Number of metastases: ≥2 vs 1 metastasis	0.6695	1.30 (0.39,4.26)
	≥2 metastases	8	4 (50.00%)	4 (50.00%)	NR (0.67,NR)					

Variables			Stratified Kaplan-Meier model				Cox regression			
	Stratum value	N	PD events (%)	Censored (%)	Median (95% CI)	Log-rank (p-value)	N	Contrast	Pr > ChiSq	Hazard Ratio (95% CI)
**KRAS mutation**	No	28	11 (39.29%)	17 (60.71%)	NR (3.17,NR)	0.8396	37	KRAS mutation: Yes vs No	0.8397	1.13 (0.36,3.54)
	Yes	9	4 (44.44%)	5 (55.56%)	20.53 (0.67,NR)					
**BRAF mutation**	No	34	14 (41.18%)	20 (58.82%)	20.53 (4.97,NR)	0.5744	37	BRAF mutation: Yes vs No	0.5796	0.56 (0.07,4.30)
	Yes	3	1 (33.33%)	2 (66.67%)	NR (9.50,NR)					
**TP53 mutation**	No	33	13 (39.39%)	20 (60.61%)	NR (5.30,NR)	0.4095	37	TP53 mutation: Yes vs No	0.4169	1.87 (0.41,8.43)
	Yes	4	2 (50.00%)	2 (50.00%)	NR (1.27,NR)					
**ICI duration**	≤4.6 months	19	11 (57.89%)	8 (42.11%)	2.73 (1.53,5.30)	0.0001	37	ICI duration: >4.6 vs ≤4.6 months	**0.0009**	0.13 (0.04,0.44)
	>4.6 months	18	4 (22.22%)	14 (77.78%)	NR (20.53,NR)					
**LDH**	≤247 U/L	21	10 (47.62%)	11 (52.38%)	15.33 (3.17,NR)	0.5002	32	LDH: ≤247 U/L vs >247 U/L	0.5031	1.49 (0.47,4.76)
	>247 U/L	11	4 (36.36%)	7 (63.64%)	NR (1.07,NR)					
**Tumoral PD-L1**	<50%	18	7 (38.89%)	11 (61.11%)	NR (2.20,NR)	0.9614	37	Tumoral PD-L1: ≥50% vs <50%	0.9615	1.03 (0.37,2.84)
	≥50%	19	8 (42.11%)	11 (57.89%)	20.53 (2.73,NR)					

PD, progressive disease; CI, confidence interval; Pr>ChiSqr, Chi-Squared test; NR, not reached; PS, Performance status. Significant p-values are highlighted in bold.

Table S2. Multivariate PFS analysis

Variable		Parameter Estimate	Standard Error	Chi-Square	Pr > ChiSq	Hazard Ratio	95% Hazard Ratio Confidence Limits	
**PD-L1 index groups**	High vs low PD-L1 index	1.47016	1.11181	1.7485	0.1861	4.350	0.492	38.447
**Sex**	Male vs Female	2.04976	1.24156	2.7256	0.0987	7.766	0.681	88.515
**ICI duration**	>4.6 vs ≤4.6 months	-1.05690	0.70383	2.2549	0.1332	0.348	0.087	1.381
**Cancer staging**	IV vs III stage	1.66037	0.94361	3.0962	0.0785	5.261	0.828	33.442

Pr>ChiSqr, Chi-Squared test. Significant p-values are highlighted in bold.

Table S3. Univariate analysis for overall survival

Variable		Stratified Kaplan-Meier model					Cox regression			
	Stratum value	N	PD events N(%)	Censored N(%)	Median (CI 95%)	Log-rank (p-value)	N	Contrast	Pr > ChiSq	Hazard Ratio (CI 95%)
**PD-L1 index groups**	High PD-L1 Index	24	14 (58.33%)	10 (41.67%)	18.03 (6.77,25.23)	**0.0348**	37	PD-L1 index: High vs Low PD-L1 Index	**0.0462**	3.72 (1.02, 13.56)
	Low PD-L1 Index	13	3 (23.08%)	10 (76.92%)	NR (1.87,NR)					
**Sex**	Female	10	1 (10.00%)	9 (90.00%)	NR (25.23,NR)	**0.0007**	37	Sex: Male vs Female	**0.0273**	30.86 (1.47, 648.28)
	Male	27	16 (59.26%)	11 (40.74%)	9.87 (4.63,25.00)					
**Age**	≤68 years	19	8 (42.11%)	11 (57.89%)	25.00 (7.53,NR)	0.5156	37	Age: >68 vs ≤68 years	0.5173	1.37 (0.53, 3.57)
	>68 years	18	9 (50.00%)	9 (50.00%)	18.03 (4.63,NR)					
**Smoker**	No	2	2 (100.00%)	0 (0.00%)	17.55 (9.87,25.23)	0.5673	37	Smoker: Yes vs No	0.5702	0.65 (0.15, 2.89)
	Yes	35	15 (42.86%)	20 (57.14%)	25.00 (8.13,NR)					
**Former smoker**	No	13	5 (38.46%)	8 (61.54%)	25.23 (6.77,NR)	0.5150	37	Former smoker: Yes vs No	0.5171	1.41 (0.50, 4.01)
	Yes	24	12 (50.00%)	12 (50.00%)	25.00 (4.97,NR)					
**Current smoker**	No	26	14 (53.85%)	12 (46.15%)	25.00 (7.53,NR)	0.3003	37	Current smoker: Yes vs No	0.3087	0.52 (0.15, 1.82)
	Yes	11	3 (27.27%)	8 (72.73%)	NR (2.40,NR)					
**Performance status (ECOG)**	0	11	5 (45.45%)	6 (54.55%)	25.00 (6.77,NR)	0.5607	37	Performance status (ECOG): 1,2 vs 0	0.5623	1.36 (0.48, 3.89)
	1,2	26	12 (46.15%)	14 (53.85%)	18.03 (4.97,NR)					
**Cancer staging**	III	12	4 (33.33%)	8 (66.67%)	25.23 (1.87,NR)	0.3298	37	Cancer staging: IV vs III	0.3359	1.74 (0.56, 5.33)
	IV	25	13 (52.00%)	12 (48.00%)	18.03 (6.77,NR)					
**Histology**	Adenocarcinoma	24	9 (37.50%)	15 (62.50%)	25.23 (12.83,NR)	0.1066	37	Histology: Adenocarcinoma vs Squamous	0.1151	0.46 (0.18, 1.21)
	Squamous	13	8 (61.54%)	5 (38.46%)	9.87 (1.43,NR)					

Variable		Stratified Kaplan-Meier model					Cox regression			
	Stratum value	N	PD events N(%)	Censored N(%)	Median (CI 95%)	Log-rank (p-value)	N	Contrast	Pr > ChiSq	Hazard Ratio (CI 95%)
**Number of metastases**	1 Metastasis	17	9 (52.94%)	8 (47.06%)	12.83 (6.77,NR)	0.8857	25	Number of metastases: ≥2 vs 1 metastases	0.8857	0.91 (0.27, 3.11)
	≥2 Metastases	8	4 (50.00%)	4 (50.00%)	25.00 (1.37,NR)					
**KRAS mutation**	No	28	14 (50.00%)	14 (50.00%)	25.00 (8.13,NR)	0.6378	37	KRAS mutation: Yes vs No	0.6392	0.74 (0.21, 2.59)
	Yes	9	3 (33.33%)	6 (66.67%)	NR (1.37,NR)					
**BRAF mutation**	No	34	16 (47.06%)	18 (52.94%)	25.00 (7.53,NR)	0.6103	37	BRAF mutation: Yes vs No	0.6143	0.59 (0.08, 4.52)
	Yes	3	1 (33.33%)	2 (66.67%)	NR (12.83,NR)					
**TP53 mutation**	No	33	15 (45.45%)	18 (54.55%)	25.23 (8.13,NR)	0.9201	37	TP53 mutation: Yes vs No	0.9201	0.93 (0.21, 4.14)
	Yes	4	2 (50.00%)	2 (50.00%)	25.00 (2.40,NR)					
**ICI duration**	≤4.6 months	19	15 (78.95%)	4 (21.05%)	6.77 (1.77,12.83)	**<.0001**	37	ICI duration: >4.6 vs ≤4.6 months	**0.0012**	0.03 (0.00, 0.26)
	>4.6 months	18	2 (11.11%)	16 (88.89%)	NR (25.23,NR)					
**LDH**	≤247 U/L	21	10 (47.62%)	11 (52.38%)	25.00 (6.77,NR)	0.4208	32	LDH: ≤247 U/L vs >247 U/L	0.4251	1.61 (0.50, 5.20)
	>247 U/L	11	4 (36.36%)	7 (63.64%)	NR (1.43,NR)					
**Tumoral PD-L1**	<50%	18	9 (50.00%)	9 (50.00%)	25.00 (8.13,25.23)	0.6714	37	Tumoral PD-L1: ≥50% vs <50%	0.6720	0.81 (0.31, 2.14)
	≥50%	19	8 (42.11%)	11 (57.89%)	NR (2.40,NR)					

PD, progressive disease; CI, confidence interval; Pr>ChiSqr, Chi-Squared test; NR, not reached; PS, Performance status. Significant p-values are highlighted in bold.

Table S4. Multivariate analysis for overall survival

Variable		Parameter Estimate	Standard Error	Chi-Square	Pr > ChiSq	Hazard Ratio	95% Hazard Ratio Confidence Limits	
**PD-L1 index groups**	High vs Low PD-L1 index	0.33590	0.70740	0.2255	0.6349	1.399	0.350	5.598
**Sex**	Male vs Female	15.63440	1731	0.0001	0.9928	6165031	0.000	.
**ICI duration**	>4.6 vs ≤4.6 months	-2.15709	1.05814	4.1558	0.0415	0.116	0.015	0.920

Pr>ChiSqr, Chi-Squared test. Significant p-values are highlighted in bold.
